# DNA Methylation and Gene Expression Profiling of Ewing Sarcoma Primary Tumors Reveal Genes That Are Potential Targets of Epigenetic Inactivation

**DOI:** 10.1155/2012/498472

**Published:** 2012-09-12

**Authors:** Nikul Patel, Jennifer Black, Xi Chen, A. Mario Marcondes, William M. Grady, Elizabeth R. Lawlor, Scott C. Borinstein

**Affiliations:** ^1^Department of Quality Control, XBiotech, Inc., Austin, TX 78744, USA; ^2^Department of Pathology, Vanderbilt University, Nashville, TN 37232, USA; ^3^Department of Biostatistics, Vanderbilt University, Nashville, TN 37232, USA; ^4^Division of Clinical Research, Fred Hutchinson Cancer Research Center, Seattle, WA 98109, USA; ^5^Department of Pathology, University of Washington School of Medicine, Seattle, WA 98195, USA; ^6^Department of Medicine, University of Washington School of Medicine, Seattle, WA 98109, USA; ^7^Department of Pediatrics and Pathology, University of Michigan, Ann Arbor, MI 48109, USA; ^8^Division of Pediatric Hematology-Oncology, Department of Pediatrics, Vanderbilt University, Nashville, TN 37232-6510, USA

## Abstract

The role of aberrant DNA methylation in Ewing sarcoma is not completely understood. The methylation status of 503 genes in 52 formalin-fixed paraffin-embedded EWS tumors and 3 EWS cell lines was compared to human mesenchymal stem cell primary cultures (hMSCs) using bead chip methylation analysis. Relative expression of methylated genes was assessed in 5-Aza-2-deoxycytidine-(5-AZA)-treated EWS cell lines and in a cohort of primary EWS samples and hMSCs by gene expression and quantitative RT-PCR. 129 genes demonstrated statistically significant hypermethylation in EWS tumors compared to hMSCs. Thirty-six genes were profoundly methylated in EWS and unmethylated in hMSCs. 5-AZA treatment of EWS cell lines resulted in upregulation of expression of hundreds of genes including 162 that were increased by at least 2-fold. The expression of 19 of 36 candidate hypermethylated genes was increased following 5-AZA. Analysis of gene expression from an independent cohort of tumors confirmed decreased expression of six of nineteen hypermethylated genes (*AXL, COL1A1, CYP1B1, LYN, SERPINE1,*) and *VCAN*. Comparing gene expression and DNA methylation analyses proved to be an effective way to identify genes epigenetically regulated in EWS. Further investigation is ongoing to elucidate the role of these epigenetic alterations in EWS pathogenesis.

## 1. Introduction

Ewing sarcoma (EWS) is a malignant tumor of bone and soft tissue that most commonly affects adolescents and young adults. EWS can be distinguished from histologically similar tumors by the presence of a characteristic chromosomal translocation that creates an abnormal fusion product that links domains from the TET and ETS protein families. The most common chromosomal translocation, present in 85% of all EWS, is t(11; 22)(q24; q12) which results in the formation of a *EWSR1-FLI1* fusion gene [1]. EWS-Fli1 and other TET-ETS fusion proteins function as aberrant transcription factors and are essential for carcinogenesis [2]. However, the EWS-Fli1 fusion protein is not sufficient by itself to promote tumor formation. As is found in many tumors, additional genetic effects are required, including mutations or gene alterations in *CDKN1A*, *TP53*, or *CDKN2A* that affect expression or protein function [2–4]. However, most EWSs do not contain these alterations. Thus, we hypothesize that uncharacterized mutations and epigenetic alterations play a key role in the development of EWS.

 The role of aberrant DNA methylation in the carcinogenesis of human malignancies is well established and has been shown to contribute to the pathogenesis of pediatric neoplasms as well as serve as molecular biomarkers that correlate with clinical behavior of these tumors [5, 6]. However, the connection between methylation and the pathogenesis of EWS has not been extensively studied. One barrier to genomewide methylation analysis for EWS is that large amounts of high-quality DNA are required for the majority of the methods for globally assessing the epigenome, which can be problematic in rare tumors. To overcome this technical issue, we used a bead array platform to characterize the methylation status of over 500 genes in DNA from formalin fixed paraffin embedded (FFPE) primary EWS. Our findings suggest that epigenetic inactivation of specific genes plays a role, at least in part, in their pathogenesis and the investigation of these alterations may lead to the identification of critical pathways that contribute to malignancy.

## 2. Materials and Methods

### 2.1. Cell Culture, hMSC Generation, and EWS Tumor Samples

EWS cell lines A673, SK-ES-1, and SK-N-MC were purchased from ATCC (Manassas, VA) and cultured per supplier's protocol. Human marrow stroma primary cultures, a representation of human mesenchymal stem cells (hMSCs), were generated from eight adult female patients and DNA was isolated as described [7]. FFPE EWS tumor specimens were acquired from the Vanderbilt University pathology archives, isolated from patients, treated from 1993 to 2009. Approval from the Vanderbilt University Institutional Review Board was granted prior to tissue acquisition or medical record review. Samples were chosen as described (see Supplemental Figure S1 available online at doi:10.1155/2012/498472). Each tissue sample was independently verified to be EWS by an experienced pathologist (J.B.) one of the authors of this paper, or K.W., (see Acknowledgements section) and found to contain 50%–90% viable tumor.

### 2.2. DNA Isolation

 EWS primary tissue was supplied as 20 *μ*M sections (1.5 × 1.5 cm) or as unstained slides containing 5 *μ*M tissue cut from FFPE tissue blocks. Genomic DNA was extracted using Instagene Matrix (Bio Rad, CA) or RecoverAll Total Nucleic Acid Isolation Kit (Life Technologies, CA) using the manufacturer's instructions and eluted in nuclease free-water. 

### 2.3. Bead Chip Methylation Analysis

DNA isolated from cell lines, hMSCs, and FFPE EWS tumor samples was quantified using PicoGreen fluorimetry (Life Technologies, Carlsbad, CA), bisulfite treated using the EZ DNA Methylation Kit (Zymo Research, Orange, CA), and eluted to achieve a prebisulfite DNA concentration of 50 ng/*μ*L. Bisulfite-treated DNA (100–250 ng) was labeled and investigated using the bead chip Methylation Cancer Panel I bead array and the data collected using a BeadArray Reader (Illumina, San Diego, CA) [8]. Each sample was run in the FHCRC Genomics Shared Resource Core, and the data was analyzed using Genome Studio Software (Version 3.2, Illumina, San Diego, CA). 37 of 52 primary ES tumors and 4 of 8 hMSC samples were run in duplicate. To minimize false positives, gender bias, and to identify biologically active hypermethylated genes, not all probe sites were used in our analysis. Of the set of 1505 CpG sites represented on the bead array, probes located in CpG islands, not on the X or Y chromosomes, and within the promoter region of the gene (defined as −700 bp to +300 bp) were included, totaling 820 CpGs in 503 genes. Raw data is shown in Supplemental Table S2, which identified 138 CpGs in 129 genes (FDR < 0.05, *P* < 0.05) that demonstrated increased methylation in primary EWS tumors.

### 2.4. Statistical Methodology

Bead chip methylation analysis, data quality control, and normalization were performed in Bioconductor package *methylumi* (http://www.bioconductor.org/). The moderated *t*-statistic implemented in Bioconductor *LIMMA* package was used to detect differentially methylated sites for cell line comparisons. This statistic has the same interpretation as standard *t*-statistic; however the standard errors were calculated to shrink towards a common value by empirical Bayes model to borrow information across all genes [9]. The *P* values from moderated *t*-tests were adjusted by Benjamini and Hochberg's method to control for false discovery associated with multiple samples [10].

 Illumina gene expression data, raw data filtering and quantile normalization were performed using the Bioconductor package *lumi*, a bead array-specific software package for Illumina microarray data. The *LIMMA *package and moderated *t*-test were also used to detect differentially expressed genes. Raw *P* values were adjusted as above to control for false discovery.

### 2.5. 5-AZA Treatment

A673, SK-ES-1, and SK-N-MC cell lines were passaged and plated at log phase. Twenty-four hours after initial passage, the media was changed to contain 2.5 *μ*M (A673 and SK-ES-1) or 100 nM (SK-N-MC) 5-AZA (stock solution 2 mM in DMSO). Media was changed every 24 hours for a total drug exposure of 120 hours. The cells were then recovered with drug-free media for 24 hours. RNA was then isolated using Trizol (Life Technologies, Carlsbad, CA) and RNeasy (Qiagen, Valencia, CA) following manufacturer's instructions. Optimal 5-AZA dosing was determined experimentally to minimize cell death while still resulting in a greater than 2-fold increase in *RASSF1A* and *CALCA* expression per TaqMan Assay (Assay nos. Hs00200394 and HS01100741, resp.) (Supplemental Figure S4). 

### 2.6. 5-AZA Expression Microarray Analysis

RNA from Mock treated and 5-AZA treated EWS cell lines was labeled using Illumina TotalPrep RNA Amplification Kits (Life Technologies, Carlsbad, CA) and analyzed in duplicate on Illumina HT-12 whole genome expression bead chips (Illumina, San Diego, CA) in the FHCRC Genomics Shared Resource Core. Data analysis was performed as described previously in statistical methodology. Probes with an FDR < 0.05 and ≥2-fold increase in expression in the 5-AZA treated samples were included in the analysis. Raw data is found in Supplemental Table S3.

### 2.7. Methylation-Specific PCR

 MSP primer pairs for *CALCA* were designed using MethPrimer for methylated (F: 5′ GAGAGTAAGATTGGAGTTCGTAGTC 3′; R: 5′ AAATAATCTCTATTAATCCGCGAT 3′) and unmethylated (F: 5′ GAGTAAGATTGGAGTTTGTAGTTGA 3′; R: 5′ CAAATAATCTCTATTAATCCACAAT 3′) genes [11] and performed on bisulfite-treated genomic DNA at an annealing temperature of 55°C for 40 cycles to generate 115 and 114 bp amplicons for methylated and unmethylated transcripts, respectively. Enzymatically methylated DNA was produced using *SssI* methylase (New England Biolabs, Beverly, MA) and used as a positive control.

### 2.8. Bisulfite Sequencing

 Bisulfite-treated DNA was PCR amplified with *LYN* and *CYP1B1 *bisulfite sequencing primers designed with MethPrimer: *LYN* (F 5′ TTTTTTAATAATATTTTGGGGATGG 3′; R: 5′ AACTTTAAAAACACAAAAACCTAAC 3′, 191 bp amplicon); *CYP1B1 *(F 5′ TTTGTAATAATTTATTTGAAGAGGT 3′; R 5′ ATAAAAACAACAAATATCCAAACC 3′, 179 bp amplicon). PCR conditions were as follows: 95° × 15′; (94° × 30′′; 55° × 30′′; 72° × 30′′) × 35 cycles; 72° × 10′. PCR amplicons were cloned into a TA vector (Life Technologies, Carlsbad, CA), transformed, subjected to DNA extraction, and sequenced. Sequencing data was analyzed using BiQ software [12].

### 2.9. Gene Expression Analysis of Primary EWS Tumors

 Total RNA was isolated from 32 primary tumor EWS biopsies obtained from Children's Oncology Group and Children's Hospital Los Angeles tumor repositories and from 3 adult primary bone marrow-derived hMSC cultures (kindly provided by Dr. D. Prockop) using Qiagen miRNA kit (Qiagen, Valencia, CA). All specimens were obtained in compliance with HIPAA regulations and following protocol review by institutional review boards. RNA was processed for whole genome expression profiling using Affymetrix GeneChip Human Exon 1.0 ST oligonucleotide microarrays according to Affymetrix protocols. Raw data (.cel) files from 10 EWS cell line samples were kindly provided by Dr. T. Triche (CHLA). The cel files for normal adult tissues were downloaded from the Affymetrix website (http://www.netaffx.com/). Data for core probeset regions from all arrays were quantile normalized using robust multichip averaging in the Partek Genomics Suite software platform (Partek, St. Louis, Mo). Transcript level data were derived from normalized exon data using median summarization, and comparison of gene expression was performed on transcript-summarized data using analysis of variance with multiple test correction.

### 2.10. Quantitative RT-PCR

 RNA was isolated from 5-AZA- or mock-treated EWS cell lines using Trizol (Life Technologies, Carlsbad, CA) and RNeasy purification (Qiagen, CA) following the manufacturer's instructions. Complimentary DNA (cDNA) was synthesized using SSII reverse transcriptase (Life Technologies, Carlsbad, CA) per reagent protocol. TaqMan assays (Life Technologies, Carlsbad, CA) for *LYN *(no. Hs00176719) and *CYP1B1* (no. Hs00164383) were performed on a Step One Plus (Life Technologies, Carlsbad, CA) or Opticon 2 thermocycler (Bio-Rad, Hercules, CA) in triplicate and normalized using *GUSB* expression (Assay no. Hs99999908). Relative expression was calculated using the comparative C_T_ method (ΔΔC_T_). Each assay was repeated three times, and error bars were generated by calculation of the standard error of the mean.

## 3. Results

### 3.1. EWS Cell Lines and Primary Tumors Demonstrate DNA Hypermethylation

**Figure 1:**
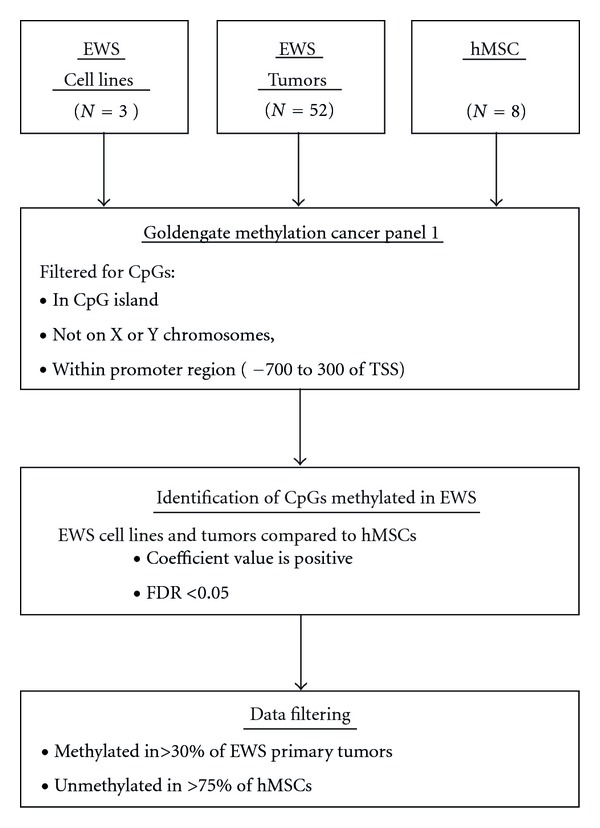
Flow chart describing workflow of DNA methylation analysis.

 The methylation profiles of three EWS cell lines and fifty-two primary tumors were characterized using bead chip methylation arrays. Tumor samples were acquired from the Vanderbilt University Pathology archives from 1993 until 2009. DNA suitable for bead chip analysis was successfully extracted from fifty-two archived samples. Sixty-two percent of the patients were male, and the average age was 19.5 years (range 3–51 years, median 16 years). Interestingly, 46% were metastatic at diagnosis. 38 (73%) samples were obtained at diagnosis, 6 (11.5%) at time of resection, and 8 (15%) at recurrence (Supplemental Table S1 and Figure S1). To assess methylation changes in EWS, the methylation patterns of these primary tumors were compared to eight bone marrow stroma primary cultures. We chose these cells as our control population as they are an accurate *in vitro* representation of hMSCs [7]. DNA from each tumor sample was analyzed using the Illumina Methylation Cancer Panel 1 array, and the majority were run in duplicate (37 of 52 primary ES tumors and 4 of 8 hMSC samples). The data obtained from duplicate samples was highly reproducible for cell lines (*R* ^2^ correlation range 0.98-0.99, mean 0.99) and FFPE tumor samples (*R* ^2^ correlation range 0.67–0.99, mean 0.90, median 0.94). Probes from the bead array were filtered to contain only CpG dinucleotides located in CpG islands, within promoter region of known genes (defined as −700 to +300 bp of the transcription start site) and excluded from the X and Y chromosome to prevent false positive results secondary to gender differences (Figure 1). 

**Figure 2:**
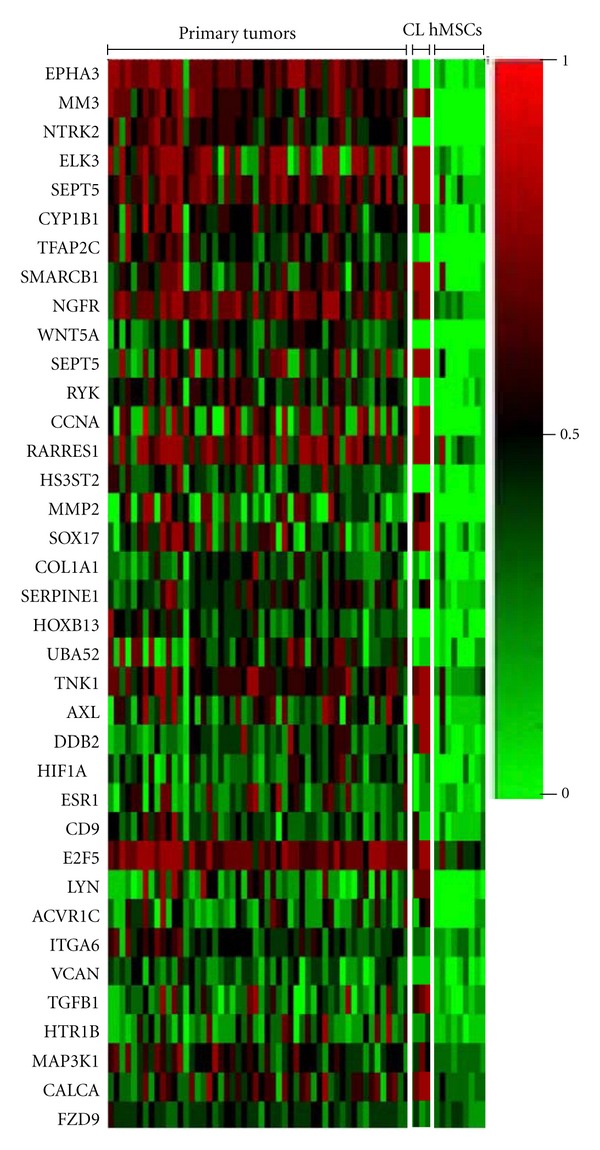
Heat map depicting DNA hypermethylation in EWS primary tumors. 37 CpGs in 36 genes were found to be hypermethylated in >30% of EWS primary tumors (*n* = 52) and >75% of hMSCs (*N* = 8). Also shown are methylation analyses of EWS cell lines A673, SK-N-MC, and SK-ES-1, respectively. Red: methylated; green: unmethylated, CL: cell lines.

 One hundred thirty-eight CpGs in 129 genes were identified as differentially methylated, defined as having a False Discovery Rate (FDR) < 0.05 as demonstrated by increased methylation in EWS primary tumors, as compared to hMSCs (Supplemental Table S2). To enrich our dataset for strongly hypermethylated genes, we further characterized several genes to assess if methylation density correlates with **β** score. Candidate genes that were found to have **β** scores ranging from 0.10 to 0.99 in EWS primary tumor samples, EWS cell lines, or hMSC primary cell cultures and cell lines were subjected to bisulfite sequencing (Supplemental Figure S2). Samples with **β** scores less than 0.40 were sparsely methylated, and samples with **β** scores greater than 0.9 were heavily methylated. We also observed that EWS primary tumors often demonstrated a heterogeneous population of completely methylated and unmethylated transcripts. We hypothesized that these results are due to normal tissue contamination of tumor samples, DNA hemimethylation, or heterogeneous clonal population in the primary tumors. Given these results, we further filtered our dataset such that a CpG probe was defined as methylated if the **β** score was greater than 0.9 for EWS cell lines and greater than 0.5 for primary tumors. A CpG was considered unmethylated in hMSC samples if the **β** score was less than 0.4. This analytic strategy yielded 37 probes in 36 genes that were hypermethylated in greater than 30% of EWS cell lines and primary tumors and were unmethylated in hMSCs (shown in Figure 2 and Table 1).

### 3.2. Validation of Bead Chip Results

**Figure 3:**
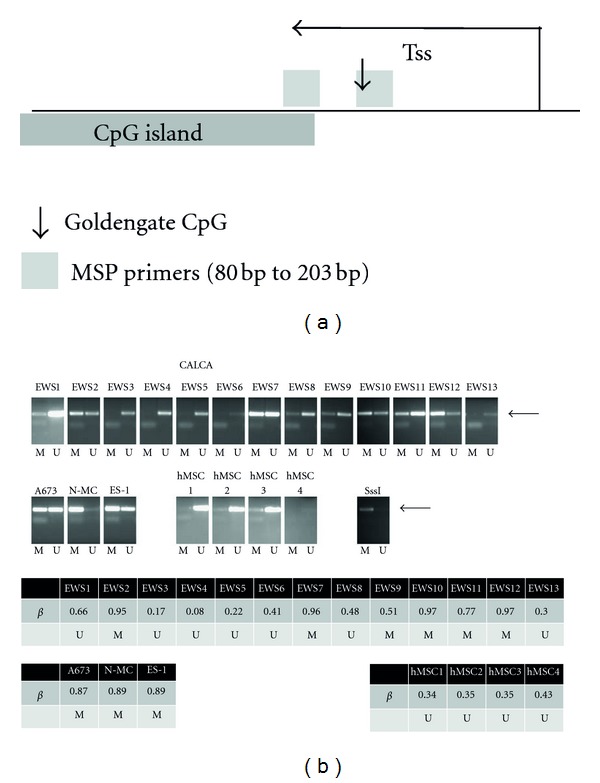
Validation of bead chip methylation analysis. (a) Schematic diagram of *CALCA* showing the location of the transcription start site (Tss) in relation to the CpG island, MSP primers, and the CpG analyzed by the bead chip assay. (b) MSP analysis of EWS primary tumors, EWS cell lines, and hMSCs. M: methylated; U: unmethylated. Bead chip methylation **β** values for EWS primary tumors EWS1–EWS13 are also shown. Tumors were considered methylated if the **β** score was greater than 0.50.

 Extensive validation of the bead chip methylation data was performed using conventional methylation analytic methodologies. As an example, MSP primers were designed for *CALCA*, a gene that has been shown to be methylated in both adult and pediatric neoplasms [13, 14]. *CALCA *was demonstrated by bead array analysis to be hypermethylated in a majority of EWS primary tumors and EWS cell lines. MSP assays were performed on 13 primary EWS tumor samples, and excellent correlation was observed when compared to the bead chip methylation **β** values (Figure 3(b)). Only tumor EWS8, which had a *β* value of 0.48 (below the cutoff value of 0.5), showed discrepancy between MSP and the bead chip results. EWS cell lines demonstrated the presence of both methylated and unmethylated alleles, which may represent hemimethylation of the *CALCA* gene or intercellular variability in the cell lines. Many other genes were analyzed in a similar fashion and also validated the accuracy of the methylation bead chip array (Supplemental Figure S2, S5, and Figure 5). 

### 3.3. Epigenetic Unmasking Reveals Genes Upregulated Upon Treatment with 5-AZA in EWS Cells

 In order to identify genes that are epigenetically silenced by DNA methylation, EWS cell lines were cultured in the presence of 5-AZA, a demethylating drug that inhibits DNA methyltransferases, for 5 days and then were allowed to recover for 24 hours (Supplemental Figure S3A). The optimal concentration to induce the expression of methylated genes and minimize nonspecific toxicity of 5-AZA was experimentally defined as that which led to a 2-fold increase in expression of *RASSF1A* or *CALCA* in EWS cell lines (Supplemental Figure S4). 5-AZA treatment of three EWS cell lines (A673, SK-ES-1 and SK-N-MC) led to the identification of 1447, 484, and 391 genes that demonstrated a 2-fold increase in expression as compared to mock-treated cells (Supplemental Figure S3B and Table S3). Importantly, one hundred sixty-two genes were significantly induced by 5-AZA in all three cell lines suggesting that either these genes are themselves epigenetically silenced by DNA hypermethylation or that their expression is secondarily modulated by 5-AZA responsive genes. Comparison of the list of 5-AZA-responsive genes to our methylation analysis shows 19 of our 36 candidate genes (53%) were upregulated downstream of 5-AZA in at least one EWS cell line treated (Table 1). 

### 3.4. Primary Tumor Microarray Analysis Suggests that Genes Are Epigenetically Repressed in EWS

**Figure 4:**
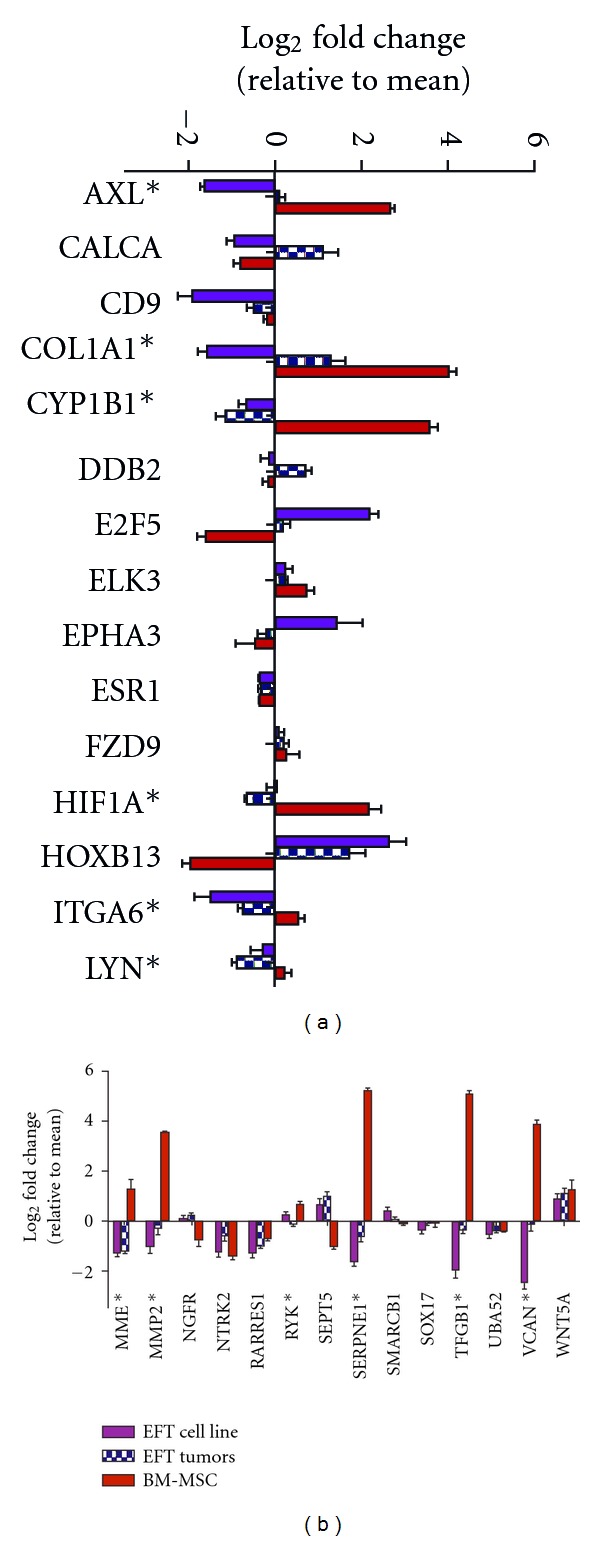
Gene expression in primary EWS tumors, cell lines, normal adult tissues, and hMSCs. Normalized Affymetrix gene expression data from 10 EWS cell lines (purple boxes), 32 EWS primary tumors (hatched purple boxes), 11 adult tissues (each in triplicate; green boxes), and 3 hMSC primary cultures (red boxes) were analyzed, and 29 of 36 hypermethylated genes were found to be differentially expressed among the 4 cell/tissue types (FDR < 0.05). Mean expression in each group is plotted relative to the mean expression level in all samples (error bars represent standard error of the mean). Asterisks denote the 12 genes that showed decreased expression in EWS primary tumors compared to hMSC (*P* < 0.05).

**Figure 5:**
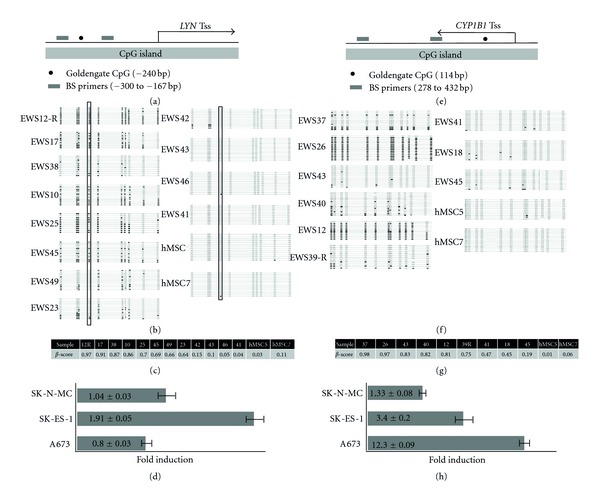
*LYN* and *CYP1B1* methylation analysis. (a) Schematic diagram of *LYN* showing the location of the Tss in relation to the CpG island, bisulfite sequencing primers, and the CpG analyzed by beadchip assay. (b) Bisulfite sequencing of *LYN* in EWS primary tumors and hMSCs. (c) Bead chip **β** scores for each sample shown. (d) qRT-PCR for *LYN* in EWS cell lines with or without 5-AZA treatment. Error bars demonstrating the standard error of the mean (SEM) are shown. (e) Schematic diagram of *CYP1B1*. (f) Bisulfite sequencing of *CYP1B1* in EWS primary tumors and hMSCs. (g) Bead chip **β** scores for *CYP1B1.* (h) qRT-PCR for *CYP1B1* in EWS cell lines with or without 5-AZA treatment.

 In order to determine if candidate hypermethylated genes are also downregulated in EWS compared to hMSC or other normal tissues, we analyzed gene expression microarray data from an independent cohort of 32 primary tumors, 10 EWS cell lines, 11 normal adult tissues (each in triplicate), and 3 primary hMSC cultures. The relative levels of expression of the hypermethylated genes were then compared among the different cell/tissue types. Expression data were available for 34 of the 36 candidate genes and the vast majority (29 of 34) showed differences in expression among the four different cell/tissue types (FDR < 0.05, Figure 4). Notably, expression of twelve genes was specifically downregulated (*P* < 0.05) in primary EWS compared to hMSCs (marked with asterisks in Figure 4) and half of these (*AXL*,* COL1A1, CYP1B1, LYN, SERPINE1, and VCAN) *showed an increase in expression following 5-AZA treatment in at least one EWS cell line (Table 1). Bisulfite sequencing of two of these genes, *LYN *and *CYP1B1, *confirmed dense methylation surrounding the promoter regions of both genes in tumors with high bead chip methylation *β* scores and sparse to no methylation in unmethylated tumors (Figure 5). Quantitative RT-PCR analysis of 5-AZA-treated EWS cell lines demonstrated increased expression following drug treatment, supporting the hypothesis that these genes are epigenetically silenced in EWS tumors (Figures 5(d) and 5(h)). Finally, although the relative levels of gene expression in primary EWS and EWS cell lines tended to be comparable relative to hMSC and normal tissues, this was not always the case (e.g.,* AXL*, *CALCA, DDB2, EPHA3, *and* RYK* in Figure 4). These findings are consistent with our observation that significant differences in methylation patterns exist between EWS tumors and cell lines (Figure 2).

## 4. Discussion

 There is considerable evidence that epigenetic mechanisms are involved in the pathogenesis of EWS. First, the EWS-Fli1 fusion protein, which functions as a transcription factor, upregulates the gene *NKX2.2* which is thought to contribute to transcriptional repression in EWS tumors by recruiting TLE corepressors, proteins that repress transcription via histone modification [15, 16]. Second, the polycomb proteins BMI-1 and EZH2 are highly expressed in EWS and are thought to be involved in downregulation of genes implicated in differentiation [17, 18]. Polycomb repressor proteins interact with many factors that regulate DNA methylation and histone modification, and they appear to play a critical role in the maintenance of the undifferentiated phenotype in embryonic stem cells through transcriptional repression of genes involved in differentiation [19]. Third, published studies have demonstrated sensitivity of EWS cell lines to 5-AZA and HDAC inhibitors [20, 21]. These studies strongly suggest that epigenetic mechanisms contribute to EWS pathogenesis.

 In this study, the methylation patterns of EWS primary tumors and cell lines were compared to hMSCs, which are thought to be the cell of origin of EWS, using methodology to characterize the methylation patterns of over 500 genes. We chose a methylation bead array platform because it is technically robust, requires a small amount of genomic DNA (as little as 100 ng), and because multiple samples can be run in parallel, thus allowing for the identification of genes commonly hypermethylated in EWS tumors for which limited FFPE tissue is available [22]. To our knowledge, this study is the first nonbiased methylation analysis published that identifies aberrantly hypermethylated genes in EWS. Thirty-six genes were found to be hypermethylated in a panel of 52 EWS tumors and unmethylated in hMSCs. However, there have been several reports that have demonstrated that DNA hypermethylation alone does not result in gene repression [23–25]. Therefore, we also assessed the levels of gene expression of methylated genes in 5-AZA- treated EWS cell lines (epigenetic unmasking experiments) and in an independent cohort of primary tumors and hMSCs. Using this multifaceted approach, we were able to successfully identify a small cohort of genes that are epigenetically silenced by DNA hypermethylation in EWS tumors. These genes are involved in many pathways that have been implicated in tumor suppression, growth signaling networks, cellular differentiation, apoptosis, tumor invasion, and other essential pathways. In particular, we identified six interesting genes that warrant additional investigation to assess their potential contribution to the pathogenesis of EWS. *SERPINE1 *is a serine protease inhibitor that involved the Akt and JAK/STAT growth signaling pathways and also has been shown to be a regulator of apoptosis [26]. Versican (*VCAN*) encodes an extracellular proteoglycan that is involved in mesenchymal to epithelial transition [27]. *AXL* is a receptor tyrosine kinase involved in the PI3 K signaling pathway [28]. *COL1A1* encodes a collagen family protein involved in extracellular matrix. This gene has been shown to be epigenetically regulated in induced pluripotent cells [29]. *CYP1B1 *is a member of the cytochrome P450 superfamily of enzymes that is involved in drug and toxin metabolism [30]. *LYN* is a member of the Src family tyrosine kinases and, interestingly, is a known oncogene that has been implicated in EWS pathogenesis [31]. Further investigation of how these genes function in EWS is warranted to understand how epigenetic dysregulation contributes to tumor changes that promote malignancy in EWS. 

 One interesting observation made in this analysis was a significant discrepancy between methylation patterns in EWS primary tumors and EWS cell lines. We identified more methylation events in EWS cell lines than primary tumors (Figure 2), which has been observed previously in other cancer types [32]. To investigate this observation, methylation changes in *RASSF1A,* a known tumor suppression gene, were characterized. MSP and bisulfite sequencing revealed dense promoter methylation in 2 of 3 EWS cell lines, but *RASSF1A *was predominately unmethylated (8 and 11% for each bead chip probe) in EWS primary tumors (Supplemental Figure S5). These results were similar to a report by Harada et al. in 2002, which did not demonstrate *RASSF1A* methylation in 8 EWS tumors [33]. However, our findings contradict a more recent report by Avigad et al. [34]. One possible explanation for this discrepancy is that the MSP primers used to investigate methylation status used by Avigad et al. are located considerably farther downstream from the transcription start site (+220 to +300) compared to our MSP primer set and the bead chip assay (−76 to +96 and +116, resp.). Regardless, these findings suggest that characterization of methylation patterns from primary tumors is essential to best understand how this epigenetic mechanism affects EWS. 

 There are several limitations to this study. First, the methylation bead array analysis only investigates 1-2 CpG dinucleotides per gene, so it is possible that genes determined to be methylated by the bead chip methylation assay do not actually demonstrate complete promoter methylation. Other genomewide methods, such as Methyl-CpG-binding domain protein sequencing (MBD-Seq), preferentially identify genes that are densely methylated and would potentially be a complimentary approach for studying global methylation patterns in EWS [35]. However, we found that the genes with high *β* methylation scores correlated well with dense methylation by conventional bisulfite sequencing analysis, so we believe that the false discovery rate will be low based on our validation studies of a subset of the genes found to be methylated using the bead chip arrays (Figure 5 and Supplemental Figure S2). Second, the FFPE primary tumor samples did contain some normal tissue. Techniques such as laser capture microdissection to enrich the amount of tumor in each sample were not used because of the need of at least 100 ng of tissue per sample. Laser capture microdissection would have limited the number of primary tumors investigated given the scant amount of available tissue. Third, the bead chip methylation assay only investigates the methylation status of 800 genes and our filtering algorithms to minimize false positives, and gender bias decreased the number of assayed genes to 500. This limited analysis prevented a comprehensive signaling pathway analyses (e.g., gene ontogeny, Ingenuity Systems Pathway, Kyoto Encyclopedia of Genes and Genomes, etc.) to identify potential therapeutic targets. Other genomewide methodologies such as Methylated CpG Island Amplification (MCA) or Comprehensive High-throughput Arrays for Relative Methylation (CHARM) have the potential to characterize the methylation profiles of more of the genome, including areas outside of the defined promoter region, such as CpG shores, which have been shown to play an important role in the epigenetic regulation of genes [36]. However, limitations to these methodologies include the requirement for large amounts of high-quality genomic DNA (1–10 *μ*g), the need for significant statistical support to analyze the array results, and high costs to analyze the samples on these arrays compared to the bead chip arrays [24, 37, 38]. In future studies, the use of the Infinium Methylation 450 K assay (Illumina, San Diego, CA), which investigates the methylation status of >485,000 CpG sites will address some of these limitations. These more comprehensive experiments will allow for a better understanding of the epigenetic changes that contribute to tumor formation and potentially will lead to the identification of novel targets for therapy. 

 In summary, we have identified genes that are aberrantly hypermethylated in EWSs and a cohort of these is downregulated in primary tumors. Further investigation of how these genes function in EWS is now warranted to understand how dysregulation of DNA methylation contributes to tumor changes that promote malignancy in EWS. 

## Supplementary Material

Supplementary Figure S1: Flow diagram of pathology samples analyzed by methylation analysis.
Supplementary Figure S2: Bisulfite treated DNA was PCR amplified with TNFRSF10A, IGFBP7, PDGFBR, SNURF, and RUNX3 bisulfite sequencing primers designed with MethPrimer (11). PCR conditions were as follows: 95° × 15'; (94° × 30”; 55° × 30”; 72° × 30”) × 35 cycles; 72° × 10'. PCR amplicons were cloned into a TA vector (Life Technologies, Carlsbad, CA), transformed, subjected to DNA extraction (plasmid mini-prep kit, Qiagen, Valencia, CA) and sequenced. Sequencing was performed either on an ABI 3730xl DNA Analyzer. Sequencing data was analyzed using BiQ software (12). Percent methylation was calculated by dividing the total number of methylated CpGs in for each gene/tissue analyzed by the total number of CpGs investigated.
Supplementary Figure S3: A.) Schematic diagram describing treatment of EWS cell lines. B.) Venn diagram demonstrating the overlap of genes upregulated > 2-fold upon treatment of 5-AZA.
Supplementary Figure S4: A.) Optimal 5-AZA dosing for cell lines SK-ES-1 and SK-N-MC were determined experimentally to minimize cell death while still resulting in a greater than 2 fold increase in RASSF1A expression as determined by qRT-PCR. 5-AZA treatment, RNA isolation, and cDNA synthesis was performed as described in Materials and Methods. qRT-PCR for RASSF1A was performed using TaqMan Assay # Hs00200394, (Life Technologies, Carlsbad, CA) on a Step One Plus thermocycler (Life Technologies, Carlsbad, CA) or Opticon 2 thermocycler (Bio-Rad, Hercules, CA) in triplicate and normalized using GUSB expression (Assay # Hs99999908). Relative expression was calculated using the comparative CT method (∆∆CT). Each assay was repeated three times and error bars were generated by calculation of the standard error of the mean. Error bars demonstrating the standard error of the mean (SEM) are shown. B.) qRT-PCR of CALCA was performed using TaqMan technology (Assay # Hs01100741, Life Technologies, Carlsbad, CA) as described above.
Supplementary Figure S5: A.) Schematic representing RASSF1A locus showing location of MSP primers, bisulfite sequencing primers, and bead chip CpG. Tss = transcription start site. B.) Bisulfite sequencing analysis of EWS cell lines. Bisulfite sequencing primers were designed using MethPrimer (F: 5' GTAGTTTAATGAGTTTAGGTTTTTT 3'; R: 5' ATCCCTACACCCAAATTTCCATTAC 3'). Filled lollipops denote methylated CpGs; empty lollipops denote unmethylated CpGs. C.) MSP analysis of EWS cell lines A673, SK-ES-1 (ES-1), and SK-N-MC (N-MC), and hMSC cell line HS-27a (27a) were determined using primers designed for methylated (F: 5' GGGTTTTGCGAGAGCGCG 3'; R: 5' GCTAACAAACGCGAACCG 3') and unmethylated (F:5' GGTTTTGTGAGAGTGTGTTTAG 3'; R: 5' CACTAACAAACACAAACCAAAC 3' ) RASSF1A. Positive (SssI treated DNA) and negative controls (WGA amplified DNA) are shown. M= methylated; U= unmethylated D.) RASSF1A methylation comparison of EWS primary tumors and cell lines.
Supplementary Table S1: Patient Demographics.
Supplementary Table S2: Raw Methylation Data after Statistical Analysis.

## Figures and Tables

**Table 1:** Genes hypermethylated in EWS primary tumors.

Symbol	Gene name	5-AZA upregulated?	Downregulated in EWS primary tumors?
*ACVR1C*	Activin a receptor, type ic	No	∗
*AXL*	Axl receptor tyrosine kinase	Yes	Yes
*CALCA*	Calcitonin/calcitonin-related polypeptide, alpha	Yes	No
*CCNA1*	Cyclin a1	Yes	NS
*CD9*	Cd9 antigen (p24)	Yes	No
*COL1A1 *	Collagen, type 1, alpha 1	Yes	Yes
*CYP1B1*	Cytochrome p450, family 1, subfamily b, polypeptide 1	Yes	Yes
*DDB2*	Damage-specific DNA-binding protein 2	No	No
*E2F5*	EF transcription factor 5, p130 binding	No	No
*ELK3*	ETS-domain protein (SRF accessory protein 2)	Yes	No
*EPHA3*	Eph receptor a3	Yes	No
*ESR1*	Estrogen receptor 1	No	No
*FZD9*	Frizzled homolog 9	Yes	No
*HIF1A*	Hypoxia inducible factor 1, alpha subunit	No	Yes
*HOXB13*	Homeobox b13	No	No
*HS3ST2*	Heparan sulfate 3-O-sulfotransferase 2	Yes	NS
*HTR1B*	5-hydroxytryptamine receptor 1B	No	NS
*ITGA6*	Integrin, alpha 6	No	Yes
*LYN*	V-yes-1 Yamaguchi sarcoma viral related oncogene homolog	Yes	Yes
*MAP3K1*	Mitogen-activated protein kinase 1	Yes	∗
*MME*	Membrane metalloendopeptidase	No	Yes
*MMP2*	Matrix metallopeptidase 2	No	Yes
*NGFR*	Nerve growth factor receptor	Yes	No
*NTRK2*	Neurotrophic tyrosine kinase, receptor, type 2	No	No
*RARRES1*	Retinoic acid receptor responder 1	Yes	No
*RYK*	Ryk receptor-like tyrosine kinase	No	Yes
*SEPT5*	Septin 5	No	No
*SERPINE1*	Serpin peptidase inhibitor, clade e, member 1 (nexin, PAI1)	Yes	Yes
*SMARCB1*	Swi/snf-related, matrix-associated, actin-dependent regulator of chromatin, subfamily b	No	No
*SOX17*	Sry (sex determining region y)-box 17	Yes	No
*TFAP2C*	Transcription factor AP gamma	Yes	NS
*TGFBI*	Transforming growth factor, beta-induced, 68 kda	No	Yes
*TNK1*	Tyrosine kinase, nonreceptor, 1	No	NS
*UBA52*	Ubiquitin A-52 residue ribosomal protein fusion product 1	Yes	No
*VCAN *	Chondroitin sulfate proteoglycan core protein 2 (versican)	Yes	Yes
*WNT5A*	Wingless-type MMTV integration site family, member 5A	No	No

∗: Gene Expression Data unknown.

NS: Not statistically significant.
