# Supplementary material for: DNA Methylation and Gene Expression Profiling of Ewing Sarcoma Primary Tumors Reveal Genes That Are Potential Targets of Epigenetic Inactivation
Source: Sarcoma. 2012 Sep 12;2012:498472. doi: 10.1155/2012/498472 (PMC3447379; doi:10.1155/2012/498472)
Supplement: Supplementary file 1 — Supplementary Figure S1: Flow diagram of pathology samples analyzed by methylation analysis. Supplementary Figure S2: Bisulfite treated DNA was PCR amplified with TNFRSF10A, IGFBP7, PDGFBR, SNURF, and RUNX3 bisulfite sequencing primers designed with MethPrimer (11). PCR conditions were as follows: 95° × 15'; (94° × 30”; 55° × 30”; 72° × 30”) × 35 cycles; 72° × 10'. PCR amplicons were cloned into a TA vector (Life Technologies, Carlsbad, CA), transformed, subjected to DNA extraction (plasmid mini-prep kit, Qiagen, Valencia, CA) and sequenced. Sequencing was performed either on an ABI 3730xl DNA Analyzer. Sequencing data was analyzed using BiQ software (12). Percent methylation was calculated by dividing the total number of methylated CpGs in for each gene/tissue analyzed by the total number of CpGs investigated. Supplementary Figure S3: A.) Schematic diagram describing treatment of EWS cell lines. B.) Venn diagram demonstrating the overlap of genes upregulated > 2-fold upon treatment of 5-AZA. Supplementary Figure S4: A.) Optimal 5-AZA dosing for cell lines SK-ES-1 and SK-N-MC were determined experimentally to minimize cell death while still resulting in a greater than 2 fold increase in RASSF1A expression as determined by qRT-PCR. 5-AZA treatment, RNA isolation, and cDNA synthesis was performed as described in Materials and Methods. qRT-PCR for RASSF1A was performed using TaqMan Assay # Hs00200394, (Life Technologies, Carlsbad, CA) on a Step One Plus thermocycler (Life Technologies, Carlsbad, CA) or Opticon 2 thermocycler (Bio-Rad, Hercules, CA) in triplicate and normalized using GUSB expression (Assay # Hs99999908). Relative expression was calculated using the comparative CT method (∆∆CT). Each assay was repeated three times and error bars were generated by calculation of the standard error of the mean. Error bars demonstrating the standard error of the mean (SEM) are shown. B.) qRT-PCR of CALCA was performed using TaqMan technology (Assay # Hs01100741, Life Techno [file 498472.f1.pptx]

## Slide 1
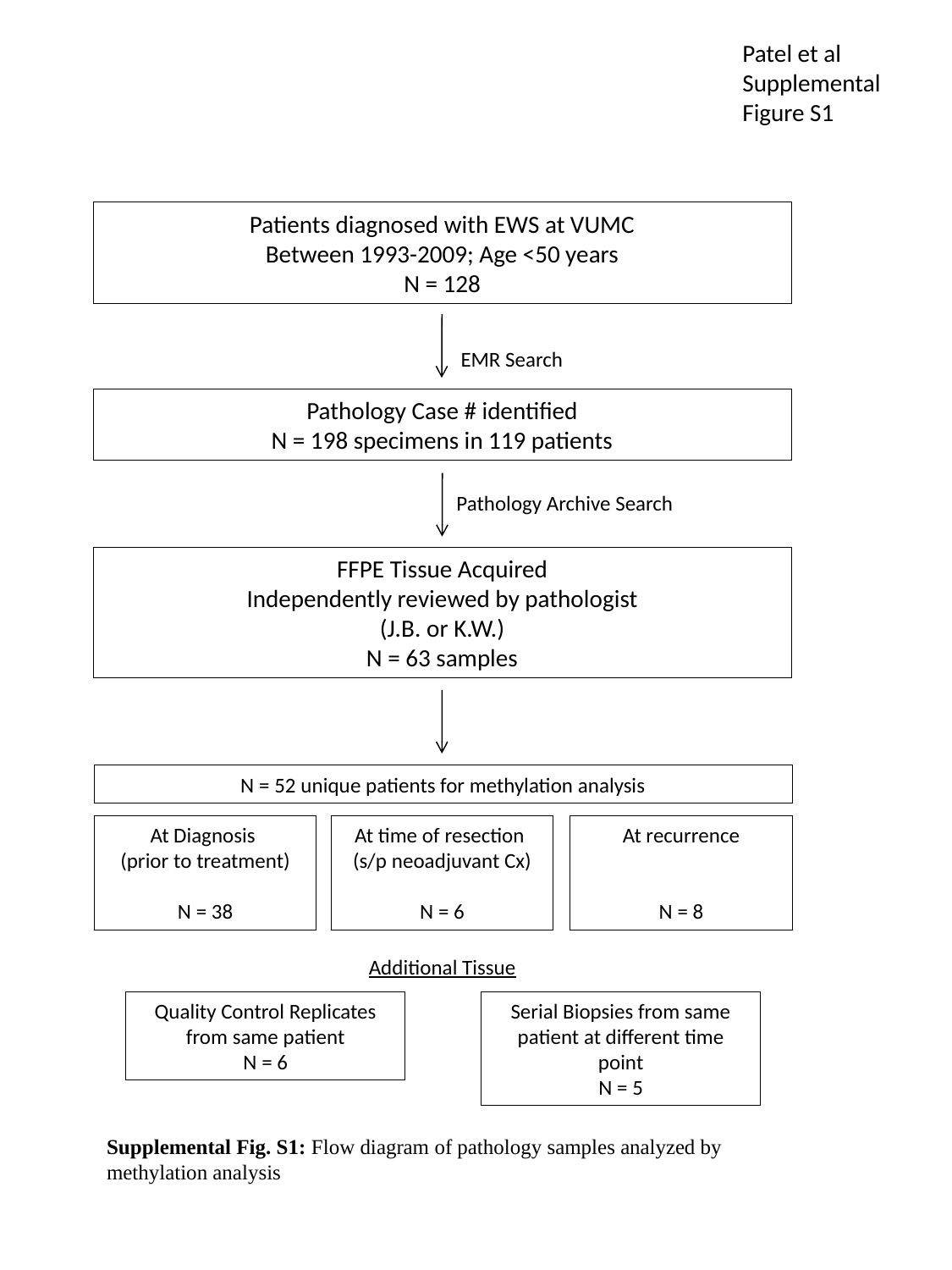

Patel et al
Supplemental Figure S1
Patients diagnosed with EWS at VUMC
Between 1993-2009; Age <50 years
N = 128
EMR Search
Pathology Case # identified
N = 198 specimens in 119 patients
Pathology Archive Search
FFPE Tissue Acquired
Independently reviewed by pathologist
(J.B. or K.W.)
N = 63 samples
N = 52 unique patients for methylation analysis
At Diagnosis
(prior to treatment)
N = 38
At time of resection
(s/p neoadjuvant Cx)
N = 6
At recurrence
N = 8
Additional Tissue
Quality Control Replicates from same patient
N = 6
Serial Biopsies from same patient at different time point
N = 5
Supplemental Fig. S1: Flow diagram of pathology samples analyzed by methylation analysis
