# Supplementary material for: DNA Methylation and Gene Expression Profiling of Ewing Sarcoma Primary Tumors Reveal Genes That Are Potential Targets of Epigenetic Inactivation
Source: Sarcoma. 2012 Sep 12;2012:498472. doi: 10.1155/2012/498472 (PMC3447379; doi:10.1155/2012/498472)
Supplement: Supplementary file 2 [file 498472.f2.pptx]

## Slide 1
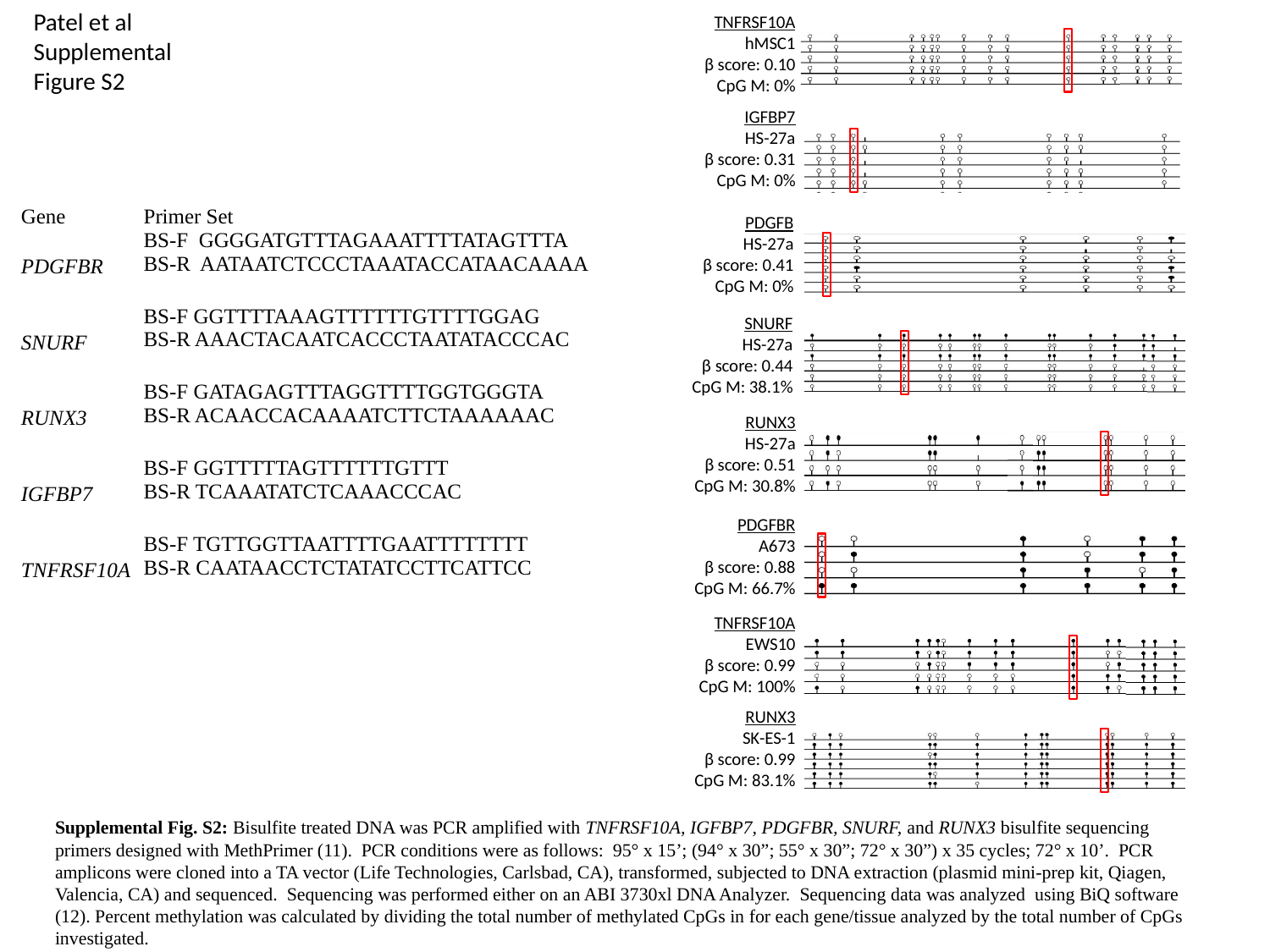

Patel et al
Supplemental Figure S2
TNFRSF10A
hMSC1
β score: 0.10
CpG M: 0%
IGFBP7
HS-27a
β score: 0.31
CpG M: 0%
| Gene | Primer Set |
| --- | --- |
| PDGFBR | BS-F GGGGATGTTTAGAAATTTTATAGTTTA BS-R AATAATCTCCCTAAATACCATAACAAAA |
| SNURF | BS-F GGTTTTAAAGTTTTTTGTTTTGGAG BS-R AAACTACAATCACCCTAATATACCCAC |
| RUNX3 | BS-F GATAGAGTTTAGGTTTTGGTGGGTA BS-R ACAACCACAAAATCTTCTAAAAAAC |
| IGFBP7 | BS-F GGTTTTTAGTTTTTTGTTT BS-R TCAAATATCTCAAACCCAC |
| TNFRSF10A | BS-F TGTTGGTTAATTTTGAATTTTTTTT BS-R CAATAACCTCTATATCCTTCATTCC |
PDGFB
HS-27a
β score: 0.41
CpG M: 0%
SNURF
HS-27a
β score: 0.44
CpG M: 38.1%
RUNX3
HS-27a
β score: 0.51
CpG M: 30.8%
PDGFBR
A673
β score: 0.88
CpG M: 66.7%
TNFRSF10A
EWS10
β score: 0.99
CpG M: 100%
RUNX3
SK-ES-1
β score: 0.99
CpG M: 83.1%
Supplemental Fig. S2: Bisulfite treated DNA was PCR amplified with TNFRSF10A, IGFBP7, PDGFBR, SNURF, and RUNX3 bisulfite sequencing primers designed with MethPrimer (11). PCR conditions were as follows: 95° x 15’; (94° x 30”; 55° x 30”; 72° x 30”) x 35 cycles; 72° x 10’. PCR amplicons were cloned into a TA vector (Life Technologies, Carlsbad, CA), transformed, subjected to DNA extraction (plasmid mini-prep kit, Qiagen, Valencia, CA) and sequenced. Sequencing was performed either on an ABI 3730xl DNA Analyzer. Sequencing data was analyzed using BiQ software (12). Percent methylation was calculated by dividing the total number of methylated CpGs in for each gene/tissue analyzed by the total number of CpGs investigated.
