# Supplementary material for: DNA Methylation and Gene Expression Profiling of Ewing Sarcoma Primary Tumors Reveal Genes That Are Potential Targets of Epigenetic Inactivation
Source: Sarcoma. 2012 Sep 12;2012:498472. doi: 10.1155/2012/498472 (PMC3447379; doi:10.1155/2012/498472)
Supplement: Supplementary file 3 [file 498472.f3.pptx]

## Slide 1
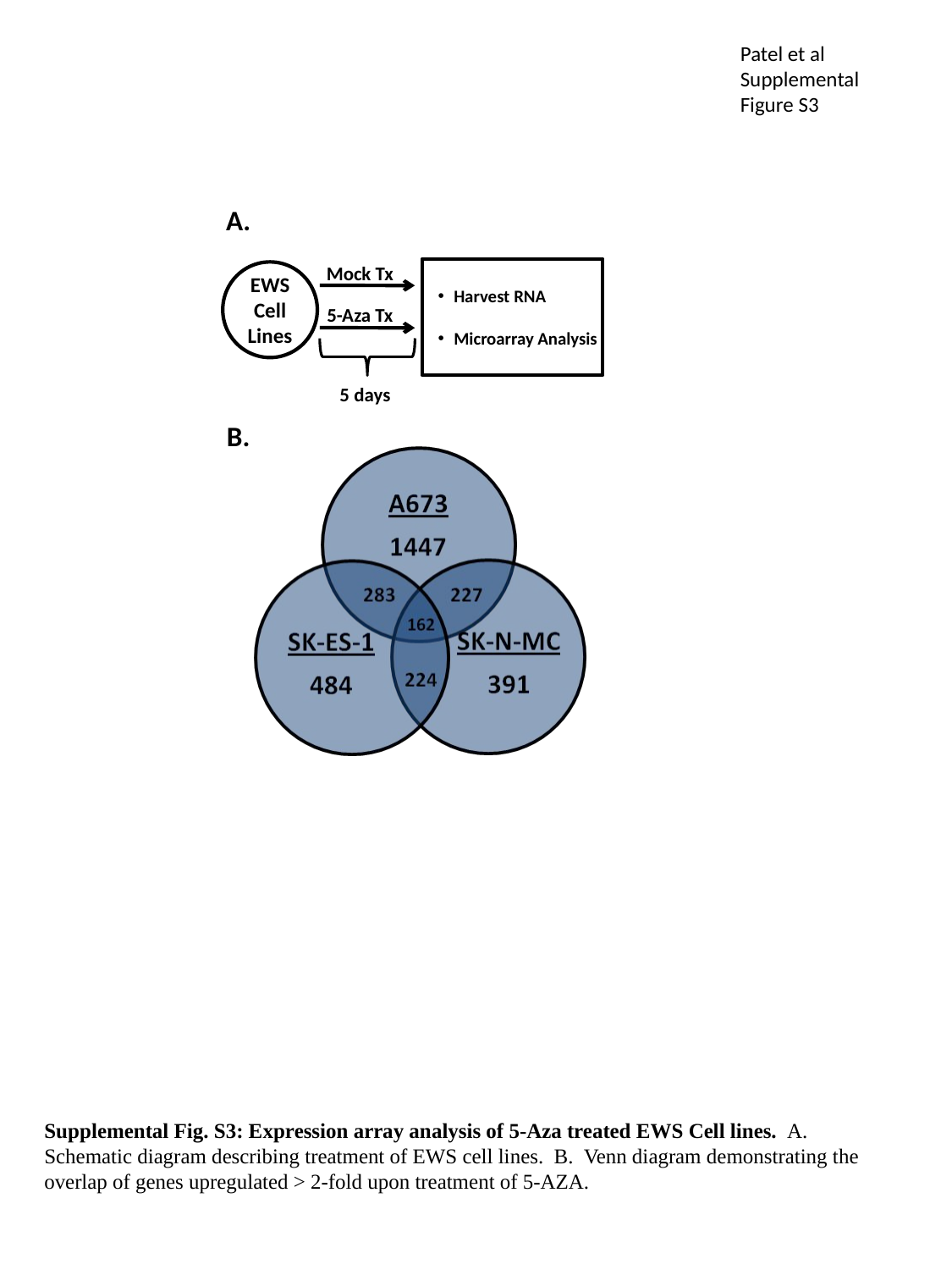

Patel et al
Supplemental Figure S3
A.
Mock Tx
Harvest RNA
Microarray Analysis
EWS Cell Lines
5-Aza Tx
5 days
B.
Supplemental Fig. S3: Expression array analysis of 5-Aza treated EWS Cell lines. A. Schematic diagram describing treatment of EWS cell lines. B. Venn diagram demonstrating the overlap of genes upregulated > 2-fold upon treatment of 5-AZA.
