# Supplementary material for: DNA Methylation and Gene Expression Profiling of Ewing Sarcoma Primary Tumors Reveal Genes That Are Potential Targets of Epigenetic Inactivation
Source: Sarcoma. 2012 Sep 12;2012:498472. doi: 10.1155/2012/498472 (PMC3447379; doi:10.1155/2012/498472)
Supplement: Supplementary file 4 [file 498472.f4.pptx]

## Slide 1
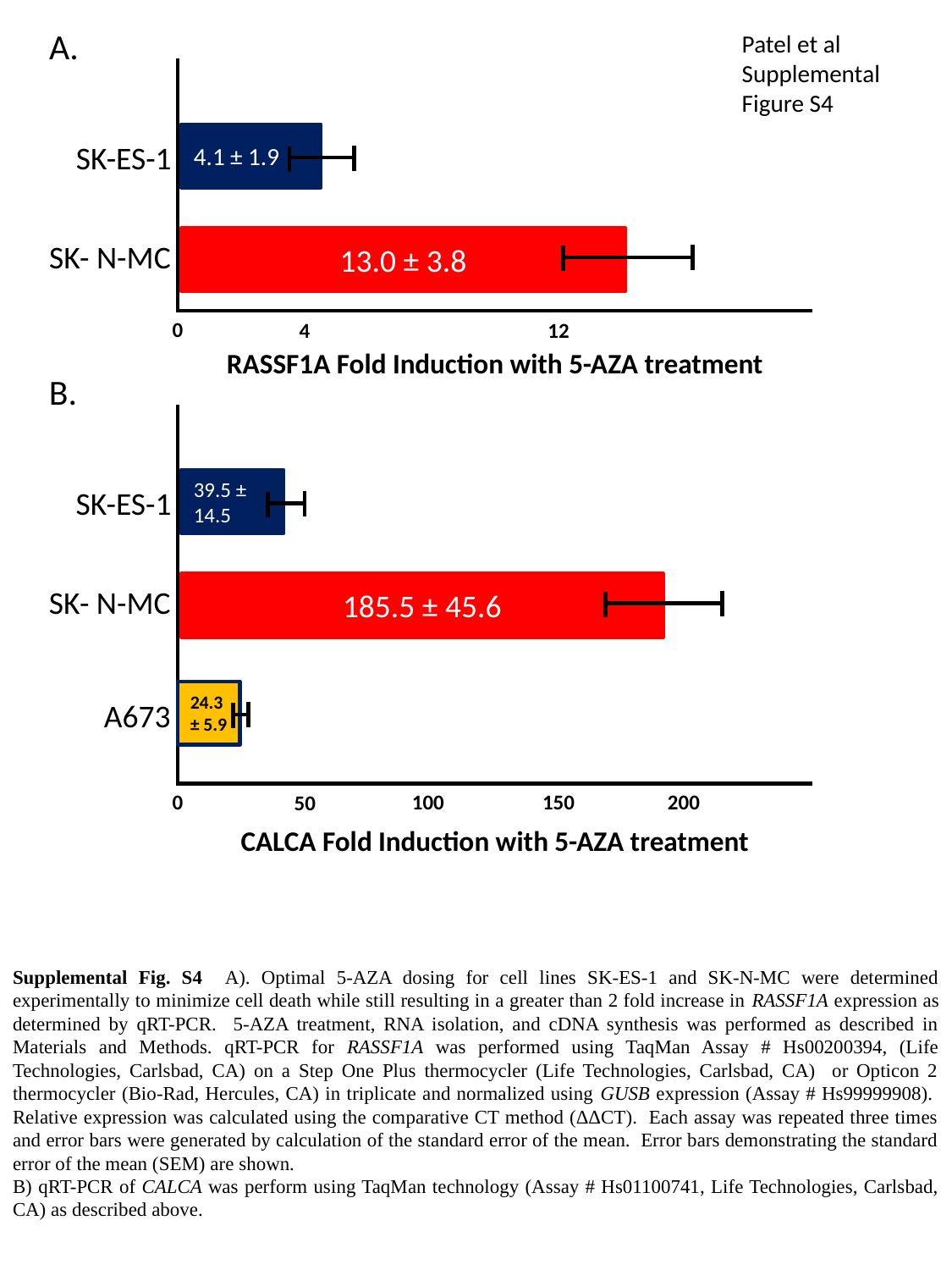

A.
Patel et al
Supplemental Figure S4
4.1 ± 1.9
SK-ES-1
13.0 ± 3.8
SK- N-MC
0
4
12
RASSF1A Fold Induction with 5-AZA treatment
B.
39.5 ± 14.5
SK-ES-1
185.5 ± 45.6
SK- N-MC
24.3 ± 5.9
A673
0
100
150
200
50
CALCA Fold Induction with 5-AZA treatment
Supplemental Fig. S4 A). Optimal 5-AZA dosing for cell lines SK-ES-1 and SK-N-MC were determined experimentally to minimize cell death while still resulting in a greater than 2 fold increase in RASSF1A expression as determined by qRT-PCR. 5-AZA treatment, RNA isolation, and cDNA synthesis was performed as described in Materials and Methods. qRT-PCR for RASSF1A was performed using TaqMan Assay # Hs00200394, (Life Technologies, Carlsbad, CA) on a Step One Plus thermocycler (Life Technologies, Carlsbad, CA) or Opticon 2 thermocycler (Bio-Rad, Hercules, CA) in triplicate and normalized using GUSB expression (Assay # Hs99999908). Relative expression was calculated using the comparative CT method (ΔΔCT). Each assay was repeated three times and error bars were generated by calculation of the standard error of the mean. Error bars demonstrating the standard error of the mean (SEM) are shown.
B) qRT-PCR of CALCA was perform using TaqMan technology (Assay # Hs01100741, Life Technologies, Carlsbad, CA) as described above.
