# Supplementary material for: DNA Methylation and Gene Expression Profiling of Ewing Sarcoma Primary Tumors Reveal Genes That Are Potential Targets of Epigenetic Inactivation
Source: Sarcoma. 2012 Sep 12;2012:498472. doi: 10.1155/2012/498472 (PMC3447379; doi:10.1155/2012/498472)
Supplement: Supplementary file 5 [file 498472.f5.pptx]

## Slide 1
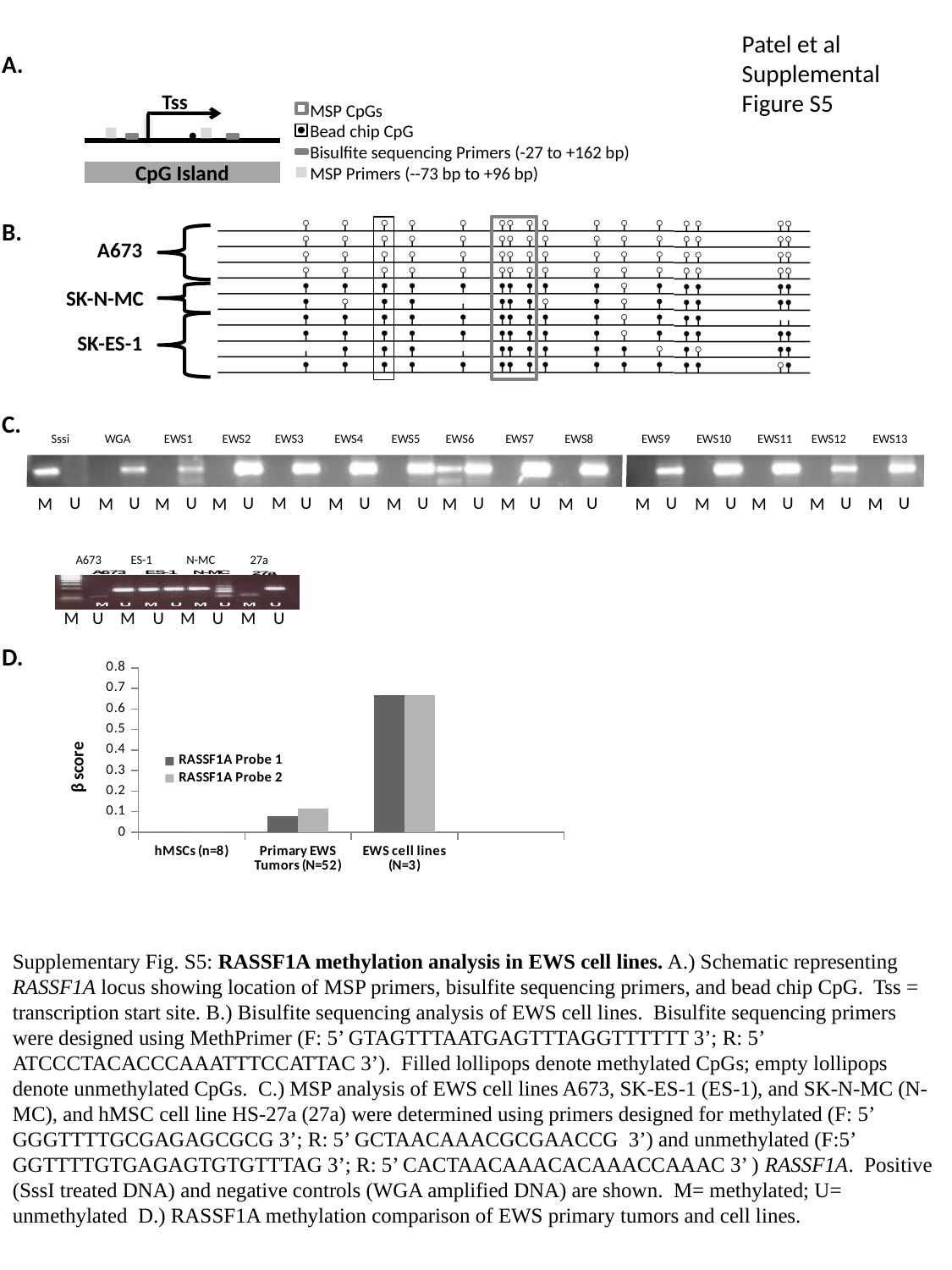

Patel et al
Supplemental Figure S5
A.
Tss
MSP CpGs
CpG Island
Bead chip CpG
Bisulfite sequencing Primers (-27 to +162 bp)
MSP Primers (--73 bp to +96 bp)
B.
A673
SK-N-MC
SK-ES-1
C.
Sssi
EWS1
EWS2
EWS3
EWS4
EWS5
EWS6
EWS7
EWS8
EWS9
EWS10
EWS11
EWS12
EWS13
WGA
M
U
U
M
U
M
U
M
U
M
U
M
U
M
U
M
U
M
U
M
U
M
U
M
U
M
U
M
U
M
A673
ES-1
N-MC
27a
M
U
M
U
M
U
M
U
D.
### Chart
| Category | RASSF1A Probe 1 | RASSF1A Probe 2 |
|---|---|---|
| hMSCs (n=8) | 0.0 | 0.0 |
| Primary EWS Tumors (N=52) | 0.07692307692307702 | 0.11538461538461549 |
| EWS cell lines (N=3) | 0.6666666666666666 | 0.6666666666666666 |Supplementary Fig. S5: RASSF1A methylation analysis in EWS cell lines. A.) Schematic representing RASSF1A locus showing location of MSP primers, bisulfite sequencing primers, and bead chip CpG. Tss = transcription start site. B.) Bisulfite sequencing analysis of EWS cell lines. Bisulfite sequencing primers were designed using MethPrimer (F: 5’ GTAGTTTAATGAGTTTAGGTTTTTT 3’; R: 5’ ATCCCTACACCCAAATTTCCATTAC 3’). Filled lollipops denote methylated CpGs; empty lollipops denote unmethylated CpGs. C.) MSP analysis of EWS cell lines A673, SK-ES-1 (ES-1), and SK-N-MC (N-MC), and hMSC cell line HS-27a (27a) were determined using primers designed for methylated (F: 5’ GGGTTTTGCGAGAGCGCG 3’; R: 5’ GCTAACAAACGCGAACCG 3’) and unmethylated (F:5’ GGTTTTGTGAGAGTGTGTTTAG 3’; R: 5’ CACTAACAAACACAAACCAAAC 3’ ) RASSF1A. Positive (SssI treated DNA) and negative controls (WGA amplified DNA) are shown. M= methylated; U= unmethylated D.) RASSF1A methylation comparison of EWS primary tumors and cell lines.
