# Supplementary material for: DNA Methylation and Gene Expression Profiling of Ewing Sarcoma Primary Tumors Reveal Genes That Are Potential Targets of Epigenetic Inactivation
Source: Sarcoma. 2012 Sep 12;2012:498472. doi: 10.1155/2012/498472 (PMC3447379; doi:10.1155/2012/498472)
Supplement: Supplementary file 6 [file 498472.f6.pptx]

## Slide 1
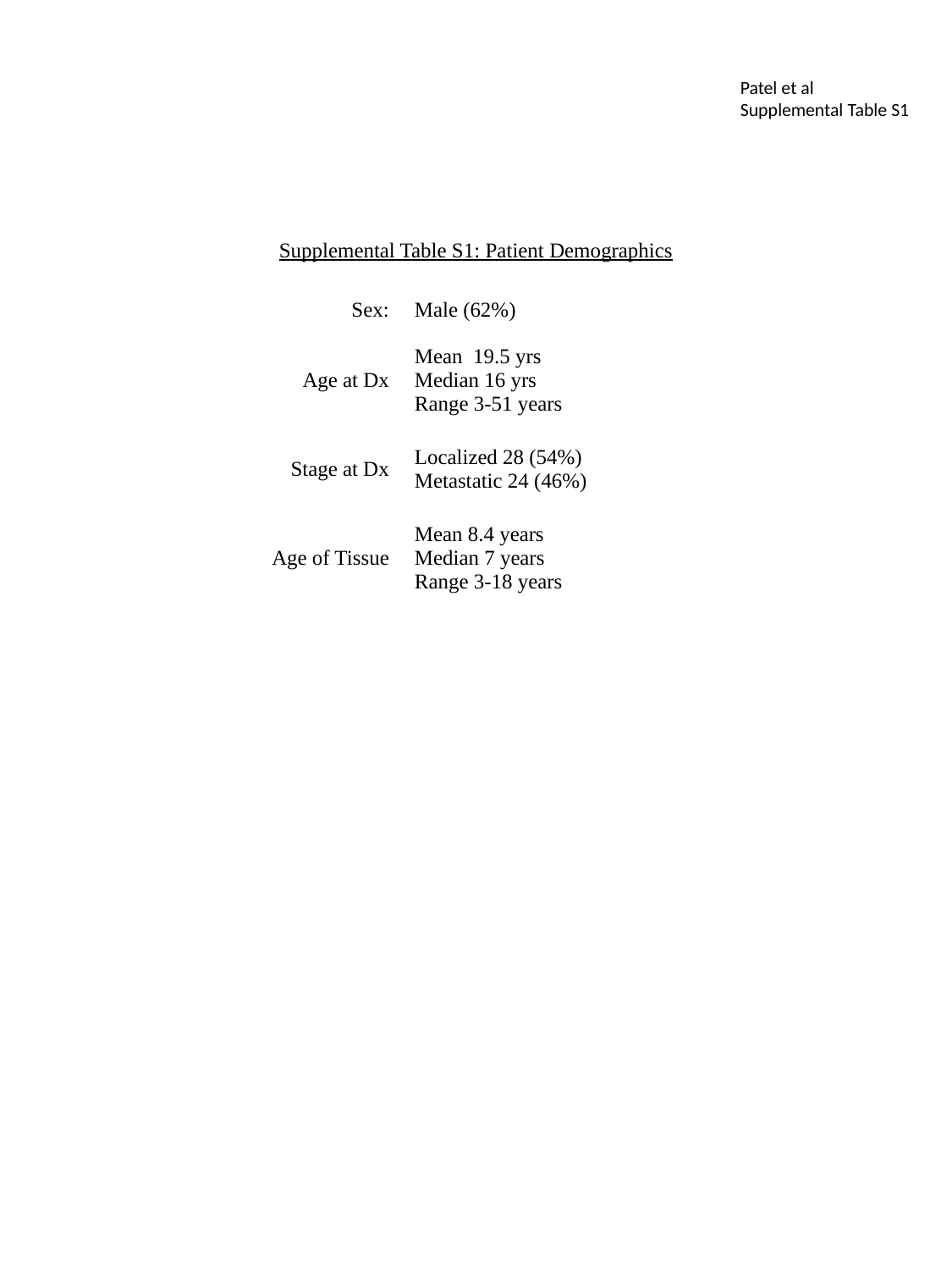

Patel et al
Supplemental Table S1
| Supplemental Table S1: Patient Demographics | |
| --- | --- |
| Sex: | Male (62%) |
| Age at Dx | Mean 19.5 yrs Median 16 yrs Range 3-51 years |
| Stage at Dx | Localized 28 (54%) Metastatic 24 (46%) |
| Age of Tissue | Mean 8.4 years Median 7 years Range 3-18 years |
